# An Overview of Alphafold's Breakthrough

**DOI:** 10.3389/frai.2022.875587

**Published:** 2022-06-09

**Authors:** Ştefan-Bogdan Marcu, Sabin Tăbîrcă, Mark Tangney

**Affiliations:** ^1^Department of Computer Science, University College Cork, Cork, Ireland; ^2^Department of Informatics, Faculty of Informatics and Mathematics, Transilvania University, Brasov, Romania

**Keywords:** AlphaFold, AlphaFold2, CASP, protein folding, AI, transformers, neural networks, deep learning

## Abstract

This paper presents a short summary of the protein folding problem, what it is and why it is significant. Introduces the CASP competition and how accuracy is measured. Looks at different approaches for solving the problem followed by a review of the current breakthroughs in the field introduced by AlphaFold 1 and AlphaFold 2.

## 1. Introduction

Proteins are the building blocks of life. They are vital to our existence with roles in almost all the biochemical processes.

**Figure 1:**
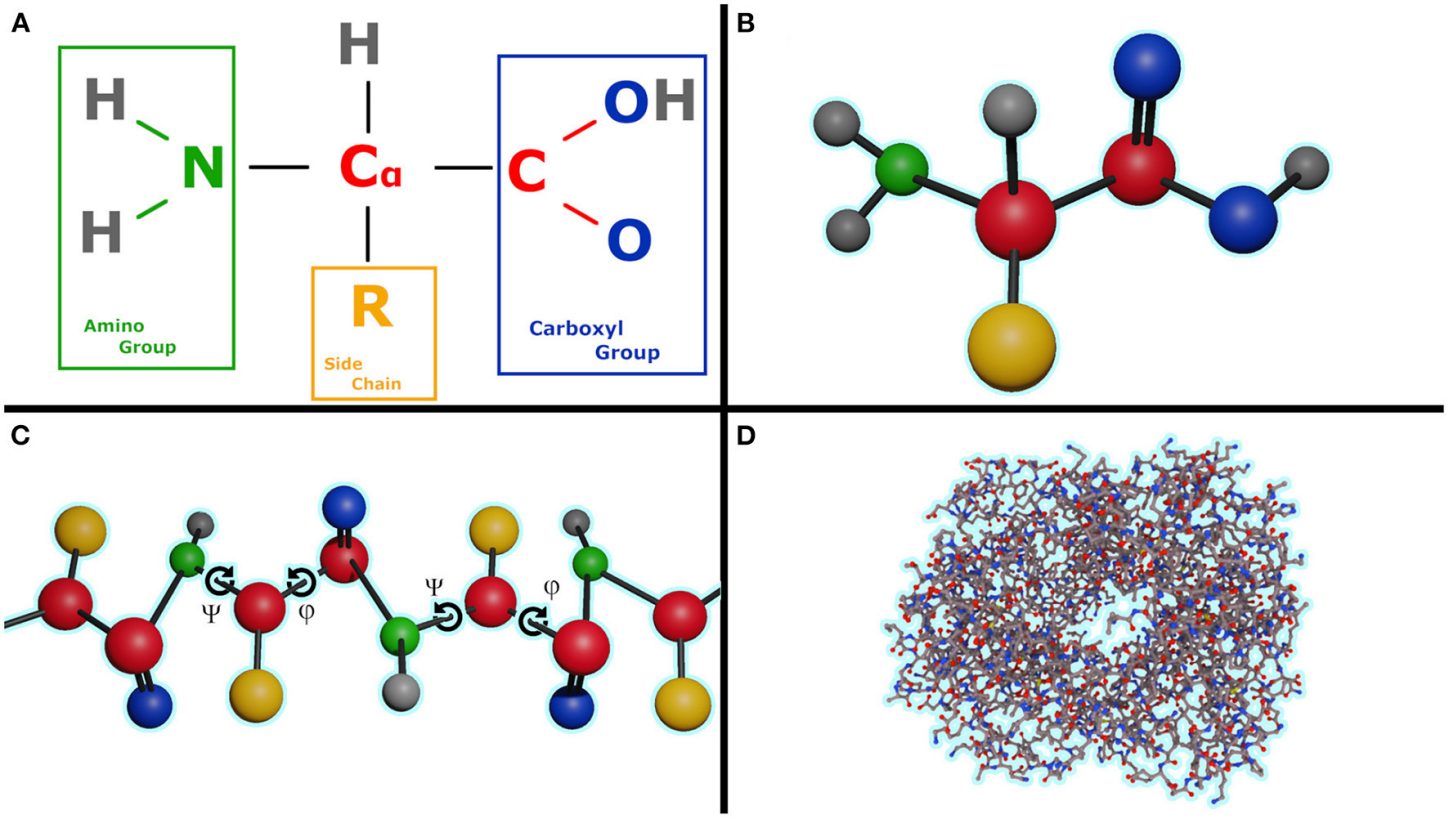
**(A)** Formula of the amino acid, **(B)** Ball and stick representation of an amino acid, **(C)** Poly-peptide chain with illustrating the torsion angles ψ and φ for each amino acid in chain, **(D)** Human hemoglobin, 1GZX, ball and chain representation with an amino acid length of 141.

A protein is made up of one or more linear chains of amino acids (a singular chain is called a poly-peptide), Figure 1B. All amino acids share a basic structure, which consists of a central carbon atom, also known as the alpha (α) carbon, bonded to an amino group (*NH*_2_), a carboxyl group (*COOH*), and a hydrogen atom. Every amino acid also has another atom or group of atoms bonded to the central atom, this is known as the residual (*R*) group, this group determines the identity of the amino acid and its chemical properties, Figure 1A. There are 20 types of amino acids commonly found in proteins.

A protein is a complex substance that consists of amino-acid residues joined by peptide bonds (Merriam-Webster, 2020), a large bio-molecule consisting of one or more long chains of amino acid residues.

### 1.1. The Importance of Protein Folding

A protein's biological function is determined by its three-dimensional native structure, which is encoded by its amino acid sequence (Jankovic and Polovic, 2017), to be biologically active, all proteins must adopt specific three-dimensional structures (Creighton, 1990). Changes in the structure of the protein can change it's behavior completely, from rendering it non-functional to rendering it toxic (Selkoe, 2003). Understanding and controlling protein folding is arguably the most important challenge in structural biology (Martnez, 2014; Jankovic and Polovic, 2017).

Denaturated proteins, which have had essentially all of their three-dimensional structure disrupted, can refold from their random disordered state into a well-defined unique structure, in which the biological activity is virtually completely restored (Levinthal, 1968). This demonstrates that they permit renaturation *in vitro*, it shows that the 3D folding structure of the protein is encoded in the sequence itself and does not depend on the creation process or on chaperones.

### 1.2. The Complexity of Protein Folding Prediction

#### The Energy Function Problem

One problem in protein folding prediction is that currently there is no easy way to calculate the exact electrostatic potential, energies of bonds slightly stretched or in non-ideal structures without involving slow and very resource intensive quantum mechanics simulations. It is crucial to know the exact value as even a small error has the potential to accumulate over time into a completely different fold. This problem is similar to the N body problem with N having an order of magnitude of 3 or higher, Figure 1D illustrates this complexity on the human hemoglobin protein.

#### The Sampling Problem

Another significant obstacle in the prediction of protein folding is the vastness of the sample space. Assuming that the bonds are of constant length, in a protein with a very modest length of 50 amino acids [the median length of a human protein is 375 (Brocchieri and Karlin, 2005)], each amino acid having two rotatable bonds in the backbone (Figure 1C angles ψ and φ), and only considering 10 degree increments we reach 100^36^) or 10^72^ possible conformations that need to be sampled in order to find the one that has the lowest energy state. The number of states is roughly equal to the estimated number of protons in the known, observable universe (136 × 2^256^≈1.57 × 10^79^ Whittaker, 1945).

### 1.3. Casp and the Protein Data Bank

CASP (Critical Assessment of Structure Prediction) is a community wide experiment to determine the state of the art in modeling protein structure from amino acid sequence organized by the Protein Structure Prediction Center. CASP is a biannual contest in which participants submit models that attempt to predict a set of proteins for which the experimental structures are not yet public (Kryshtafovych et al., 2019).

Submissions are compared with experimental results by independent assessors. The experiment is double blinded, participants have no access to the experimental structures and assessors do not know the identity of those making the submissions (Kryshtafovych et al., 2019).

The Protein Data Bank is the single worldwide archive of structural data of biological macro molecules (Berman et al., 2000). It is a leading global resource for experimental data providing access to 3D structure data for large biological molecules (PDB, 2021).

CASP and The Protein Data Bank are the enablers of the current breakthroughs in protein folding. CASP offering an impartial metric of success while the Protein Data Bank offers the data required for the training of the statistical models. Without them, the current breakthroughs would be impossible.

### 1.4. How to Measure Accuracy

The Global Distance Test or GDT has been used since CASP 4 as a metric function defining the quality of the fold prediction. GDT finds the largest subset of model residues that can be superimposed on the corresponding residues in the reference structure within a specific distance threshold (Chen and Siu, 2020). The Global Distance Test Total Score (GDT_TS) or average GDT score represents the average GDT scores with a cutoff point of 1, 2, 4, and 8Å. The values of the score range from 0 (complete mismatch) to 100 (perfect superimposition). A drawback of the of this metric is the dependence of the score magnitudes on the evaluated protein's size (Zhang and Skolnick, 2004).

## 2. Prediction Techniques

### 2.1. Physics Simulations

Based on simulating quantum mechanics, these constituted some of the first attempts in predicting the folding of a protein. The main idea in here being to rely on molecular dynamics simulations in order to obtain the protein folding. While this approach is accurate for proteins of limited size (up to hundreds of residues) the computational cost rises exponentially with the length of the protein chain. Another disadvantage of this approach is that it can get stuck due to erroneous force-fields producing inaccurate results.

It is believed that these approaches can go beyond documented structures and capture novel folds (Surbhi et al., 2020).

### 2.2. Fragment Assembly

Models are built from short, contiguous backbone fragments taken from proteins of known structure. Fragment selection is typically guided by sequence similarity, as well as by predictions of local structural features such as secondary structure or backbone torsion angles (Kuhlman and Bradley, 2019). Multiple assemblies of fragments are generated and then evaluated using a coarse-grained energy value function. For the lowest valued candidates a more fine grained, and thus more computationally expensive, value function is used in order to select the best candidate for prediction. Variations of this approach have been the best predictors in the FM category for both CASP9 (Moult et al., 2011) and CASP10 (Moult et al., 2014), and were among the two dominant tools in CASP11 (Moult et al., 2016).

### 2.3. Machine Learning

Machine-learning techniques have a long history of applications to protein structure analysis (Kuhlman and Bradley, 2019). Initially used as components in a workflow, to predict 1D features such as backbone torsion angles or secondary structure (Kuhlman and Bradley, 2019), they have recently seen a boom in the field with end-to-end approaches (Surbhi et al., 2020). Works like End-to-End Differentiable Learning of Protein Structure by Mohammed AlQuraishi (AlQuraishi, 2019b) and later the AlphaFold architectures created by DeepMind (Senior et al., 2020) showing the true predicting power of machine learning.

Successful strategies from image processing and natural language processing, such as convolutional neural networks and the attention mechanism have been incorporated in the architectures designed to predict the correct fold of a protein.

## 3. AlphaFold

### 3.1. What Is AlphaFold

AlphaFold is Alphabet's DeepMind entry in the CASP13 competition. It is an artificial intelligence system designed to tackle the problem of protein folding. A a co-evolution dependent workflow (AlQuraishi, 2019a) that has a deep convolutional neural network as its main component. This system takes the sequence for which a folding is desired to be found together with Multiple Sequence Alignment (MSA) statistics as inputs and outputs a structure prediction (Senior et al., 2020). AlphaFold takes advantage of the observation that residues in spatial contact tend to show patterns of correlated mutations (Kuhlman and Bradley, 2019).

### 3.2. CASP13 Results

AlphaFold represents an anomalous leap in protein structure prediction (AlQuraishi, 2019a). AlphaFold is able to predict more FM domains with high accuracy than any other system participating in CASP13, particularly in the 0.6–0.7 TM-score range (Senior et al., 2020).

Alpha Fold achieved a summed z-score of 52.8 compared with 36.6 for the next closest group. Furthermore combining FM and TBM/FM categories, AlphaFold scored 68.3 compared with 48.2 despite using only FM techniques (Senior et al., 2020).

### 3.3. AlphaFold Architecture

The AlphaFold solution is composed of two stages. A convolutional neural network that takes as an input the protein sequence and a series of Multiple Sequence Aligned features and outputs a protein specific potential surface, followed by a second stage consisting of multiple Gradient Descent runs in order to find the structure that best minimizes the protein potential function.

#### Deep Convolutional Neural Network

With the help of distograms (histograms showing inter-residue distances) the problem of finding the 3D structure of the protein can be interpreted as a computer vision problem. By doing so the tools and findings from computer vision can be applied to the protein folding problem. One of the pioneers of this method within the CASP competition being RaptorX-Contact server (Wang et al., 2017).

The generation of the distogram was done using a deep residual network. The input is composed of features extracted from similar protein sequences and used MSAs to generate profile features. These features were passed to a module consisting of 220 blocks of of dimension 64 x 64. Each block consists of a sequence of neural network layers that interleave three batchnorm layers; two 1 x 1 projection layers; a 3 x 3 dilated convolution layer and exponential linear unit nonlinearities (Senior et al., 2020). The network outputs a predicted distance probability over 64 equal bins representing the range between 2 and 22 Åfor each amino acid pair, allowing to not only predict a distance but also a confidence level. It also outputs a prediction for the backbone torsion angles.

The network was trained on the Protein Data Bank (PDB) structures to predict the distances *d*_*ij*_ between the *c*_β_ atoms of pairs, ij, of residues of a protein (Senior et al., 2020).

#### Gradient Descent on Protein Specific Potential

The second stage of the AlphaFold solution consists of the construction of a smooth protein specific potential filed over which an optimal minimum is calculated by Gradient Descent. In order to construct the potential field, a spline is fitted to the negative log probabilities over the bins of the distogram. The spline, parametrised by the torsion angles outputed by the neural network have been integrated into a differentiable model of protein geometry. In order to correct for the overrepresentation of the prior, a reference distribution was subtracted from the potential. To combat steric clashes a smoothing score that incorporates van der Waals forces has been added to the potential (Senior et al., 2020). The torsion angles resulted from the neural network are further refined using gradient descent on the potential function created.

The optimisation process is repeated for multiple sampled initialisations, and a genetic algorithm is applied, adding noise to the backbone torsion angles as mutation, in order to find the structure with the lowest potential.

### 3.4. Convolutional Neural Networks and Protein Folding

The potential drawback of using convolutional neural networks is that they may not map accurately over the problem. Convolutinal neural networks are invariant to translations, this makes them great at detecting an object in the picture, but not so well suited at detectinos where the positioning in the sequence conveys meaning, such as understanding a statement. The protein folding problem seems to be closer to the latter rather than the former.

A second possible drawback in using convolutional neural networks is that their information is local (generally a detection of an object is not be affected by something in the other corner of the image). This is generally good behavior in detecting images but might not take advantage of all the information available for the protein folding problem.

Another possible drawback of AlphaFold is choosing the distogram as the main representation of a proteins structure. The space of possible protein foldings is very small compared to the space of possible distograms. Because the network isn't constrained in any way to only distograms of possible protein foldings, it is possible for it to output distograms that are impossible in the real world. This greatly increases the search space that the network explores and therefore decreases its accuracy and increases the training time. Choosing the distogram as a final output of the network instead of an actual 3D structure can also be considered a bottleneck as some of the more complex and subtle interactions that the model might have learned could not be expressed using this representation, this is worsened by the fitting of a spline over the predicted distances, discarding most of the subtleties of the model.

## 4. Alphafold 2

AlphaFold2 is the second iteration of the AlphaFold system. It is DeepMind's entry in the CASP14 competition, an end to end solution for predicting a protein folding given its amino acid sequence.

### 4.1. Results

#### CASP14

During the CASP14 competition, AlphaFold2 has achieved what can be considered a breakthrough in the problem of protein folding. AlphaFold2 has achieved outstanding results not only when compared with the previous years results but also when compared to the other competing groups. DeepMind's solution has vastly outperformed the 145 other solutions participating in the CASP14 competition (Flower and Hurley, 2021) with a summed z score of 244.0217 and an average Z score of 2.6524, the closest competitor achieved a summed z score of 92.1241 with an average Z score of 1.0013 (Center, 2020). Nearly two-thirds of the predictions were comparable in quality to experimental structures (Flower and Hurley, 2021) with some cases being virtually indistinguishable from the experimental results (Flower and Hurley, 2021).

#### Outside CASP

AlphaFold2 has results outside the CASP competition as well.

AlphaFold2 prediction have been validated against the structure of ORF3a where the results have ended up being similar to structures later determined experimentally despite their challenging nature and having very few related sequences (Flower and Hurley, 2021).

### 4.2. Architecture

AlphaFold 2 is an end to end solution (Jumper et al., 2020). This not only allows the network to better fine tune by evaluating on the actual structure instead of an intermediary step. It also frees the network to explore different avenues by not having to restrain itself to an imposed strategy.

An important component of the model is a version of the iterative SE(3)-Transformer (Jumper et al., 2020). A transformer is an auto encoder architecture specialized in sequence to sequence mapping. It employs an attention mechanism that allows it to learn correlations in the input data (Vaswani et al., 2017). The Iterative SE(3)-Transformer is a graph transformer with a customized attention mechanism designed to be equivariant under continuous 3D roto-translations (Fuchs et al., 2021). By using this architecture the network no longer needs to spend resources learning that the interactions are invariable with respect to the global position and rotation.

The attention mechanism (Jumper et al., 2020) of the transformer provides a greater flexibility to the network, it dynamically learns the information flow allowing complex interaction with non-neighboring nodes. This allows the network to learn what relations are relevant and which can be ignored.

AlphaFold 2 Takes a protein sequence as input. Starting from the sequence, similar sequences are found using MSA and the raw sequences are embedded. Feeding the raw MSA sequences allows a more complex understanding of the amino-acid correlations, complexity further enriched by the attention mechanism that sifts through the data and prioritizes the most significant information. Added to these are a set of potential templates for the MSA sequences.

Because only geometrical constrains are enforced in the network, after the prediction a relaxation step is needed in order to enforce the stereo-chemical constraints (Jumper et al., 2020).

### 4.3. Equivariance and Protein Folding

Equivariance is a form of symmetry for functions from one space with symmetry to another. One example of a well known network architecture that illustrates the property of equivariance over the operation of translation is the Convolutional Neural Network. Through the embedding of the equivariance property in the model the network learns faster the concept of invariance, a more specific version of equivariance, facilitating the decoupling of an objects classification form its position in the input space. SE(3), the special Euclidean group of 3D space, extends the property of equivariance over continuous translation and rotation.

In order to help the neural network find the correct folding, the query sequence is augmented with MSA and pairwise features. These statistics are invariant under the proteins global location and it's rotation. This makes the SE(3)-Transformer a good contender as a model architecture.

### 4.4. Improvements Over AlphaFold 1

AlphaFold 2 uses an embedding that contains the full sequences found by MSA and potential templates (Flower and Hurley, 2021), while the previous solution was receiving only MSA statistics. This allows AlphaFold2 a richer more complex understanding of the protein space.

Embedding the physical and geometric notions into the architecture instead of a search process (Jumper et al., 2020). This considerably restricts the search space and allows better performance by not exploring, or even considering, physically impossible configurations.

Replacing the convolutional neural network with an attention based architecture, this replaces the rigid information flow from the local neighbors of the convolutional networks with a flow dynamically controlled by the network (Jumper et al., 2020).

Applying the model in an iterative manor using the Iterative SE(3)-Transformer allows the gradients to propagate through the whole architecture eliminating the disconnect between pairwise distance predictions and the 3D structure encountered in the initial AlphaFold model.

### 4.5. Limitations of AlphaFold 2

One limitation of approaches based on MSAs, such as AlphaFold2, is that they are constrained by our current knowledge and data sets. Such approaches are able to interpolate between known points in the protein-structure space and maybe even extrapolate around such known points but are not able to confidently and accurately predict novel configurations. I believe that in order to model such novel configurations a molecular dynamics components is essential.

Another limitation stems from the fact that AlphaFold2 was trained on PDB, this specializes the network in predicting structures as they would be found in the PDB, which may not be the natural fold state of that protein. Some of the PDB folds only happen in known special conditions, such as the presence of another protein, these special conditions are usually documented during the solving of the fold. In such a case AlphaFold2 will predict the folding as it is found in the PDB, but is unable to provide the special conditions in which the particular fold happens. This limitation is most clearly observable for proteins with multiple native structures.

## 5. Conclusion

AlphaFold 1 proved that neural networks posses the complexity required in order to be capable of modeling the protein folding mechanism. AlphaFold 2 further improves accuracy by using a more representative internal representation and embedding equivariance knowledge in the model. This frees the network from having to learn the equivariance concept and concentrate on the underlying folding mechanism.

A giant leap forwards in the problem of protein folding. AlphaFold 2 will have a big impact in the industry, enabling a less time consuming and more accessible protein folding prediction. This has the potential of accelerating the rate of discoveries in any field in which proteins play a significant role.

AlphaFold 2 illustrates the power of focusing the learning process by restricting the search space and embedding knowledge in the model.

## Author Contributions

Ş-BM wrote the manuscript with support from ST supervisor and MT co-supervisor. All authors contributed to the article and approved the submitted version.

## Conflict of Interest

The authors declare that the research was conducted in the absence of any commercial or financial relationships that could be construed as a potential conflict of interest.

## Publisher's Note

All claims expressed in this article are solely those of the authors and do not necessarily represent those of their affiliated organizations, or those of the publisher, the editors and the reviewers. Any product that may be evaluated in this article, or claim that may be made by its manufacturer, is not guaranteed or endorsed by the publisher.
